# Solitary Intrathyroid Metastasis Occurring 23 Years after Resection of Renal Cell Carcinoma

**DOI:** 10.1155/2021/2735256

**Published:** 2021-09-04

**Authors:** X. Vandemergel

**Affiliations:** Department of Endocrinology, EpiCURA, Boussu, Belgium

## Abstract

A case of solitary intrathyroid metastasis is described in a 60-year-old male patient. He had a history of renal cell carcinoma classified as T1b resected 23 years earlier. A mass was palpable in the right thyroid lobe. Ultrasound showed a hypoechoic polylobular nodule with intense vascularisation in the right lobe. Fine needle aspiration cytology was normal, but thyroidectomy was performed due to mass enlargement, the ultrasound pattern, and the oncological history. Histological examination revealed the presence of an intrathyroid metastasis of renal cell carcinoma. The bone scan and thoracoabdominal CT scan were normal. Postoperative care was uneventful.

## 1. Introduction

The four main types of primary thyroid cancer are papillary, follicular, medullary, and anaplastic carcinomas, and they represent the majority of newly diagnosed thyroid cancers [1]. The thyroid gland is a rare site of tumour metastasis. Metastatic thyroid tumours account for 1.4–3% of all thyroid neoplasms, are most often related to renal cell carcinoma (RCC) [2], and are frequently asymptomatic. Fine needle aspiration (FNA) is used for the cytological diagnosis of neoplastic lesions in most cases, but they may mimic a poorly differentiated thyroid cancer. We report here the case of a patient with a history of RCC 23 years before presentation, complaining of a palpable nodule in the right lobe. The nodule was classified as EU-TIRADS 5 based on ultrasound. FNA cytology revealed a benign nodule, but due to the ultrasound findings and the oncological history, a surgical procedure was performed. Histology revealed a metastasis of RCC. The patient gave written informed consent. A literature review was also conducted.

## 2. Case Report

A 60-year-old man was referred to our department of endocrinology for the evaluation of a neck mass. His past medical history included ischemic cardiomyopathy, hypertension, and hyperlipidaemia, and he underwent right nephrectomy 23 years earlier for RCC classified as stage 1 (T1b).

He smoked 20 cigarettes/day, and his treatment included statin, salicylic acid, angiotensin-converting enzyme inhibitor, and diltiazem.

**Figure 1:**
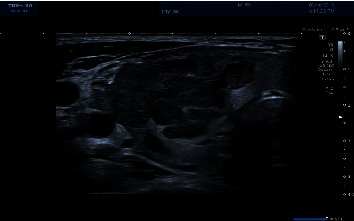
Thyroid ultrasound: linear probe and high frequency. Right lobe: hypoechoic polylobular nodule with intense intranodular vascularisation classified as EU-TIRADS 5.

In July 2019, he complained of a right cervical mass. Ultrasound (Figure 1) revealed a large hypoechoic polylobular nodule in the right lobe with a dense central vascularisation, classified as EU-TIRADS 5. FNA cytology was performed and showed normal cells and was classified as Bethesda 2.

The patient was lost to follow-up and came back in July 2020 with the same symptoms. There was no hoarseness. On clinical examination, the thyroid was enlarged and a hard nodule was palpable in the right lobe, without cervical adenopathy. Ultrasound found the same nodule measuring 42 × 22 × 28 mm. There were no microcalcifications.

Biologically, the thyrotropin level was at 0.42 mU/L, the free T4 level was normal, and there were no thyroperoxidase antibodies. The thyroglobulin level was normal at 27 pg/L.

**Figure 2:**
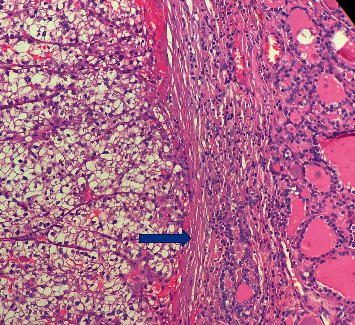
Hematoxylin-eosin staining showing large clear cells. A thin fibrovascular network is shown (arrow). Cells have an eosinophilic foamy vacuolated cytoplasm.

He underwent total thyroidectomy. The histopathological examination showed large clear cells positive for CD10, vimentin, CK19, and PAX 8 with lymphatic and venous metastasis (Figure 2). Thyroid Transcription Factor 1 (TTF1) was completely negative, ruling out a primary tumour of the thyroid.

The postoperative course was uneventful, and bone scintigraphy and computed tomography also revealed the absence of any other metastases. External radiation completed the treatment. Radiation was delivered to a total dose of 50 Gy in 25 fractions at 2 Gy per day 5 days per week.

Six months later, the patient remained free of recurrence.

## 3. Discussion

Intrathyroid metastases (ITM) of RCC are very rare despite the fact that RCC are hypervascularised tumours associated with multiple arteriovenous shunts leading to the subsequent growth of metastases through the vascular route and that the thyroid is the second most vascularised organ after the adrenal gland [3]. While clinical ITM are rare, their prevalence in autopsied series varies between 1.25 and 24.4% in patients who died from primary or metastatic malignancies [4]. After the diagnosis of RCC, the risk of recurrence is higher in the first three years, and the local recurrence-free survival rate 15 years after surgery and the distant metastasis-free survival rate at 15 years have been estimated at 95% and 86%, respectively [5]. The risk factors associated with a late recurrence are the tumour stage, the tumour size, and RCC with clear cell histology. The most common sites of metastases are the lung, bone, and liver [6].

When present, thyroid metastases are often secondary to RCC. In a retrospective surgical series including 11 patients with thyroid metastases, Dequanter et al. found that they originated from an RCC in 2 cases, from a pulmonary squamous cell carcinoma in 5 cases, and from an oesophageal, oropharynx, or breast carcinoma and a leiomyosarcoma in 1 case each [7]. In 25 cases of ITM, Calzolari et al. [2] found that they originated from an RCC in 15 cases (60%) followed by lung and colonic cancers.

The symptoms include a palpable thyroid nodule, but ITM is often an incidental finding [2]. ITM may appear many years, or even decades, after the primary lesion, and times to onset ranging between a few months and more than 27 years have been reported [8].

Kobayashi et al. [9] studied the ultrasonographic features of metastatic RCC to the thyroid. Ten patients were included (4 men and 6 women) with a mean age at the time of thyroid surgery of 67 years. The primary site was the right kidney in 9 cases. The metastatic tumour was located in the right thyroid lobe in 9 cases and in the left lobe in 3 cases (two metastases in 2 cases). The tumour shape was irregular and well demarcated with a hypoechoic solid pattern without a capsule-like structure and direct contact with the normal thyroid tissue in all cases, classically classified as EU-TIRADS 5. There were no calcifications. The intratumour vascularity was intense in 8 cases. In our case, we found the same features. Also, while microcalcifications are rarely described, they may be present [10].

In most series, FNA cytology allowed diagnosing a neoplastic lesion in most cases. In the study by Calzolari et al. [2], FNA cytology revealed the presence of neoplastic cells in 8 out of 17 patients. Case reports have focused on the positivity of FNA cytology for the diagnosis of cancer [10–13]. In a series published by Dequanter, ten patients underwent preoperative FNA and the cytological results suggested a metastatic disease in nine of these patients. Bula et al. [14] assessed the usefulness of FNA cytology for the diagnosis of tumour metastases to the thyroid gland in 733 patients with thyroid cancer (metastatic tumours in 10 cases, 7 of them of RCC origin). In all cases, FNA cytology was abnormal and showed a follicular tumour in 3 cases, tumour cells in 2 cases, and atypical cells in the remaining patients. In the study by Kobayashi et al. [9], FNA cytology showed a metastatic carcinoma of RCC in 4 cases, an indeterminate nodule in 2 cases, a benign nodule in 3 cases, and a follicular neoplasm in 1 case for a total of 10 FNA performed. FNA cytology was strictly normal in our case, showing that despite normal FNA cytology, patient history and the ultrasound appearance of the lesion should be taken into account in the surgical decision making.

The 5-year overall survival of patients with ITM varies greatly depending on whether a single metastasis is present and only located in the thyroid or whether multiple metastases are present. In the study by Calzolari et al. [2], 50% of patients had a single ITM while 50% had multiple ITM. The actuarial 5-year overall survival was 31% (median: 48 months). The median survival of patients with multiple lesions was 30 months, and that of patients with a single metastasis in the thyroid gland was 60 months. Also, while the 5-year survival of patients with a single lesion was 48%, none of the patients with multifocal cancer was alive at 5 years.

Treatment is based on surgery. It is not known whether total thyroidectomy is always needed or if partial thyroidectomy may be useful in the management. Radiotherapy may be used for the treatment of RCC metastases [15] despite the fact that RCC have been shown to be radioresistant. It may be indicated in case of painful bone metastases, brain metastases, and painful recurrence in the renal bed [16, 17]. The usefulness of radiation is not established, but some case report concluded that irradiation may be effective at higher level of radiation [18–20].

In conclusion, we reported the case of a 60-year-old patient experiencing an isolated intrathyroid metastasis 23 years after resection of a right-sided renal cell carcinoma. Despite the usefulness of FNA cytology, the ultrasound pattern and the clinical history should be taken into account in the surgical decision making.

## Data Availability

The data are available in the archives of the hospital.
